# Multispecies mass mortality of marine fauna linked to a toxic dinoflagellate bloom

**DOI:** 10.1371/journal.pone.0176299

**Published:** 2017-05-04

**Authors:** Michel Starr, Stéphane Lair, Sonia Michaud, Michael Scarratt, Michael Quilliam, Denis Lefaivre, Michel Robert, Andrew Wotherspoon, Robert Michaud, Nadia Ménard, Gilbert Sauvé, Sylvie Lessard, Pierre Béland, Lena Measures

**Affiliations:** 1 Maurice Lamontagne Institute, Fisheries and Oceans Canada (DFO), Mont-Joli, Québec, Canada; 2 Canadian Cooperative Wildlife Health Centre, Faculté de Médecine Vétérinaire, Université de Montréal (UM), St. Hyacinthe, Québec, Canada; 3 National Research Council of Canada (NRCC), Biotoxin Metrology, Halifax, Nova Scotia, Canada; 4 Environment and Climate Change Canada, Canadian Wildlife Service (CWS), Québec, Québec, Canada; 5 Groupe de Recherche et D’éducation sur les Mammifères Marins (GREMM), Tadoussac, Québec, Canada; 6 Parks Canada, Saguenay-St. Lawrence Marine Park, Tadoussac, Québec, Canada; 7 Canadian Food Inspection Agency (CFIA), Québec, Québec, Canada; 8 St. Lawrence National Institute of Ecotoxicology (SLNIE), Knowlton, Québec, Canada; University of Connecticut, UNITED STATES

## Abstract

Following heavy precipitation, we observed an intense algal bloom in the St. Lawrence Estuary (SLE) that coincided with an unusually high mortality of several species of marine fish, birds and mammals, including species designated at risk. The algal species was identified as *Alexandrium tamarense* and was determined to contain a potent mixture of paralytic shellfish toxins (PST). Significant levels of PST were found in the liver and/or gastrointestinal contents of several carcasses tested as well as in live planktivorous fish, molluscs and plankton samples collected during the bloom. This provided strong evidence for the trophic transfer of PST resulting in mortalities of multiple wildlife species. This conclusion was strengthened by the sequence of mortalities, which followed the drift of the bloom along the coast of the St. Lawrence Estuary. No other cause of mortality was identified in the majority of animals examined at necropsy. Reports of marine fauna presenting signs of neurological dysfunction were also supportive of exposure to these neurotoxins. The event reported here represents the first well-documented case of multispecies mass mortality of marine fish, birds and mammals linked to a PST-producing algal bloom.

## Introduction

The paralytic shellfish toxins (PST) associated with paralytic shellfish poisoning (PSP) are potent neurotoxins produced by natural, environmentally-driven populations of some marine dinoflagellates, mainly by *Alexandrium* spp [1]. PST include saxitoxin (STX) and at least 21 derivatives that can be produced by the algae in various combinations and concentrations. Grazers, such as copepods, could play a key role in the increase of paralytic shellfish toxin production by dinoflagellates [2–4]. Some of these compounds are highly neurotoxic, acting as sodium channel-blocking agents restricting signal transmission between neurons, particularly in mammals, birds and fish—with a toxic potency up to 100-fold greater than sodium cyanide [1]. Mass mortalities of farmed fish during episodic dinoflagellate blooms, the accumulation of PST in shellfish during these events and the resulting regulation for human health and their economic consequences, are well documented [5–7]. Reports of marine wildlife mortalities resulting from PST-producing algal blooms are, in contrast, unexpectedly rare [8, 9] and often anecdotal, although it is suspected that several cases have been missed or unreported due to lack of adequate investigations [10, 11]. As with other kinds of contaminants, the finding of microalgal toxins in waters or organisms alone does not necessarily imply a direct cause and effect relationship with fauna mortalities. Among the suspected important effects of PST exposure on wildlife is its potential role in the decline of endangered species such as the North Atlantic right whale *Eubalaena glacialis* [12, 13] and the shortnose sturgeon *Acipenser brevirostrum* [14] populations inhabiting New England coastal waters.

Filter-feeding aquatic organisms, such as bivalves and zooplankton, appear relatively tolerant to PST and hence can accrue high levels of these toxins by directly feeding on algae [15, 16]. This has been identified as a potential mechanism by which toxins are transferred through the food web to higher trophic levels. A classic case of transfer of PST up the food web and subsequent mortality at a higher trophic level involved humpback whales in New England, USA [8]. During a 5-week period beginning in late November 1987, 14 humpback whales, *Megaptera novaeangliae*, died in Cape Cod Bay after eating Atlantic mackerel, *Scomber scombrus*, containing PST.

In Eastern Canada, as well as in many regions of the globe, the toxic dinoflagellate *Alexandrium tamarense* has been identified as a major source of PST [17]. Like many other PST-producing algal species, this species presents a complex life cycle with a dormant phase in the sediments and a vegetative phase in the water column. After a period of dormancy, the sedimentary cysts germinate into vegetative cells that migrate to surface waters and can potentially initiate a new bloom when environmental (physical, chemical and biological) conditions are favorable. The St. Lawrence Estuary is well recognized for the high abundance of *A*. *tamarense* cysts in sediments and the recurrence of blooms of this species [18–20].

During the summer of 2008, an intense red tide of *A*. *tamarense* occurred in the St. Lawrence Estuary, which coincided with unprecedented mass mortalities of marine fish, birds and mammals. Here, we describe this multispecies mass mortality event and present a line of evidence showing that toxins produced by *A*. *tamarense* was responsible for mortalities. The present paper also provides unique information on trophic transfers, the accumulation and biotransformation of PSP toxins through the food web from plankton to marine mammals and birds, the transplacental transfer of toxins as well as neurological dysfunctions and clinical signs associated with PST intoxication. It is the first well-documented case of multispecies mass mortality of marine fish, birds and mammals linked to a PST-producing algal bloom.

## Results and discussion

**Fig 1:**
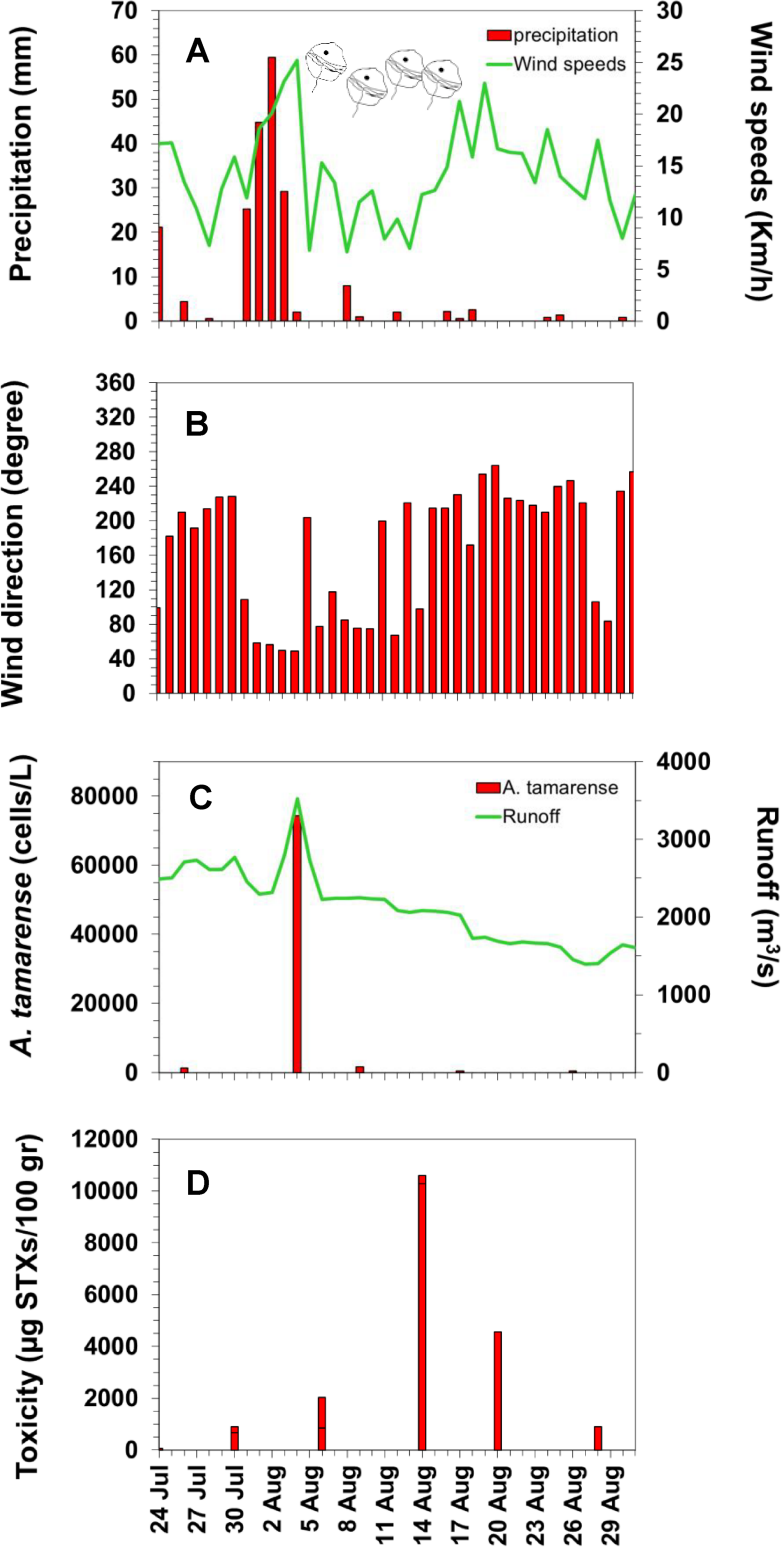
Environmental conditions associated with *A*. *tamarense* bloom in the SLE. (**A)** Daily Tadoussac rainfall (mm) (bars) and Mont-Joli airport wind speeds (solid line) and (**B)** wind direction (degree). (**C)** *A*. *tamarense* cell abundances at Tadoussac (cells L^–1^) (bars) and Saguenay River runoff (m^3^ s^–1^) (solid line). (**D)** Mollusc toxicity near Bic Island. *A*. *tamarense* cell symbols indicate the period of the bloom. See Fig 2 for the geographical positions.

**Fig 2:**
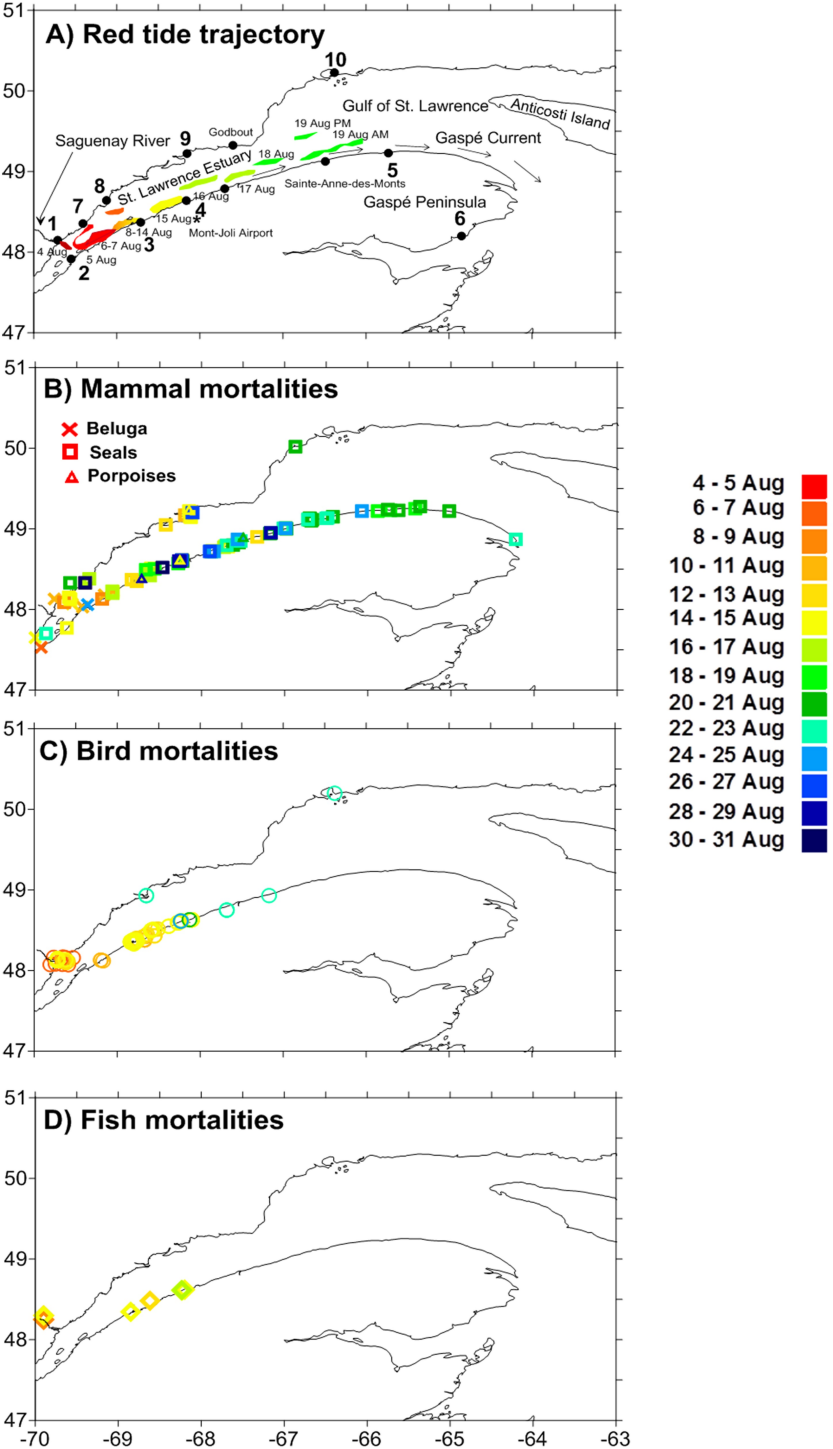
Location of shellfish and toxic algae monitoring stations and fauna mortalities. **(A)** Map of the Estuary and Gulf of St. Lawrence, Canada, showing the location of mollusc and/or toxic algae monitoring stations (numbered from Site 1 to 10), the general summer surface circulation pattern (arrows) and the algal bloom trajectory as simulated by modelling (polygons colour-coded with the date event). (**B** to **D)** Position of mammal (X: beluga; square: seals; triangle: porpoises), bird (circle), and fish (diamond) mortality events colour-coded with the event date. For bird and fish mortalities, each symbol may represent several carcasses of the same species. Site 1: Tadoussac, 2: Cacouna, 3: Bic Island, 4: Sainte-Flavie, 5: Mont-Louis, 6: Port-Daniel, 7: Escoumins, 8: Portneuf, 9: Baie Comeau, 10: Sept-Îles.

Following heavy precipitation (>130 mm within 4 days) and high river runoff (Fig 1), we observed an intense bloom of the toxic dinoflagellate *Alexandrium tamarense* in the St. Lawrence Estuary (SLE) in August 2008, which coincided with an unprecedented mass mortality of marine fish, birds and mammals (Fig 2). This typical association between *A*. *tamarense* and river runoff has been previously attributed to the beneficial effects of low salinity and high temperature on cellular growth rate, the riverine input of terrestrially-derived dissolved organic matter, nutrients and other materials such as humic substances that can serve as growth stimulants and/or increased water column stability that favours the proliferation and retention of cells [21]. However, the net population growth rate such as observed at the mouth of the Saguenay River during the high river runoff (0.75 d^-1^, Fig 1) was well above the range of growth (0.3–0.5 d^-1^) measured in laboratory studies for this species [22–24]. In addition to growth, the sudden increase in *A*. *tamarense* cell concentrations associated with high river runoff could be also due to resuspension and germination of cysts from sediments. Indeed, the SLE is recognized for the presence of high concentrations of cysts in sediments, especially where depth is less than 100 m and close to the river plumes where cyst concentrations can exceed 200 cysts cm^-3^ [20]. Moreover, vertical migration and convergent circulation can combine to accumulate cells [25]. Thus, the sudden increase in *A*. *tamarense* cell concentrations associated with high river runoff and low salinity during the month of August 2008 may thus be the result of a combination of biological and physical processes. Based on helicopter surveys, this August bloom attained some 600 km^2^ in size and remained in the SLE for two weeks before dissipating.

**Fig 3:**
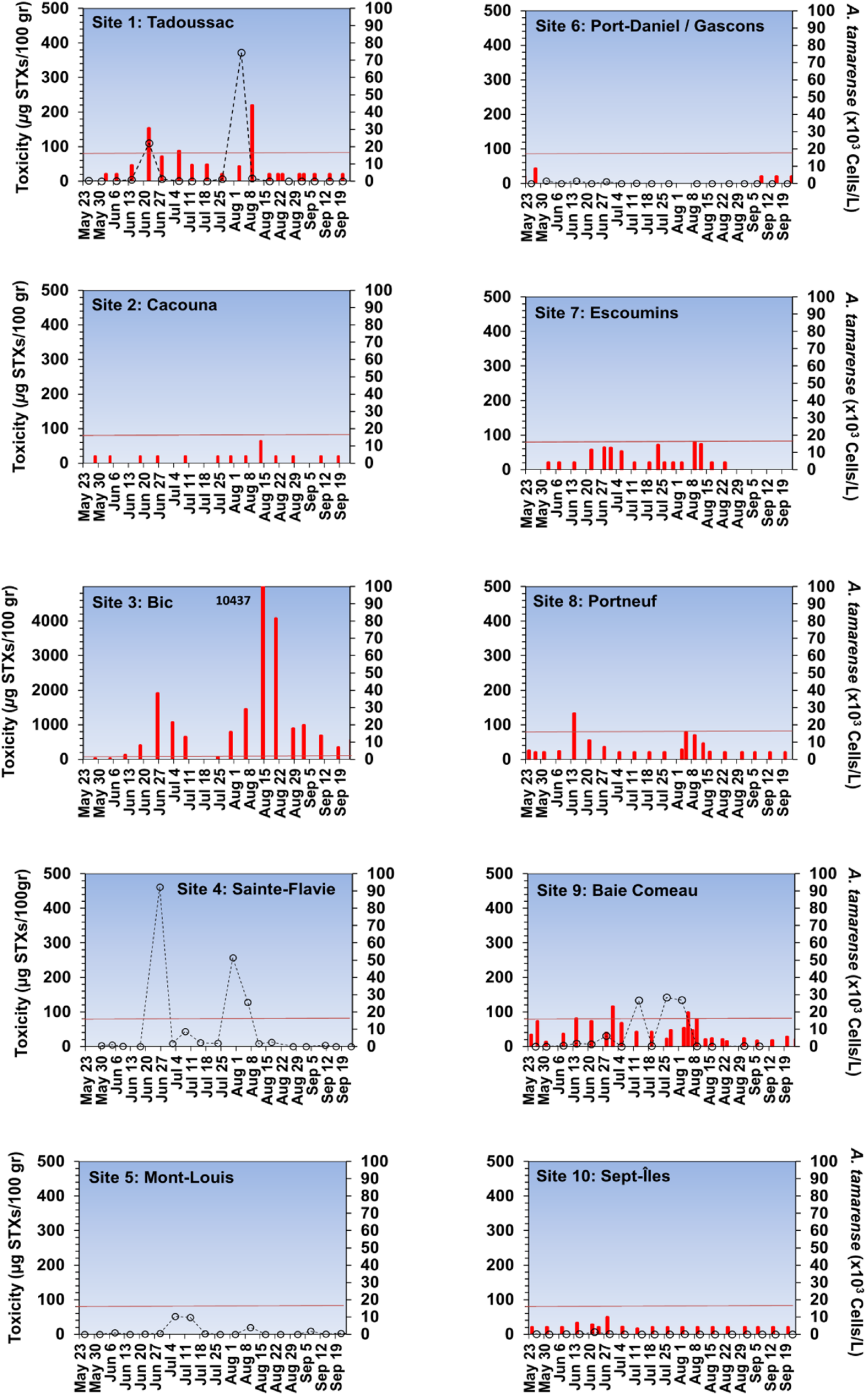
Shellfish toxicity and toxic algae monitoring data. Mollusc toxicity (red bars) and/or the abundance of *A*. *tamarense* (open circles) recorded at 10 selected monitoring sites in the Estuary and Gulf of St. Lawrence (see Fig 2). The horizontal red lines indicate the level of toxicity considered hazardous for human consumption.

The *A*. *tamarense* bloom trajectory shown in Fig 2A was simulated using observed winds and modelled currents. The position of the bloom was forecasted daily during the event to guide sampling and to warn shellfish collectors and producers. Projections were validated from mollusc toxicity and/or the abundance of *A*. *tamarense* recorded at several coastal monitoring sites in the SLE and Gulf of St. Lawrence (GSL) (Fig 3).

Up to 80 x 10^3^ cells L^-1^ of *A*. *tamarense* were first identified on August 4, 2008 at Tadoussac (Fig 1), at the confluence of the Saguenay and St. Lawrence rivers (Fig 2A, Site 1). Smaller localized increases in *A*. *tamarense* abundance and shellfish toxicity were also observed at the same time at Baie-Comeau, close to the mouth of the Manicouagan and Aux-Outardes rivers (Fig 3, Site 9). Prior to the August event, close examination of Fig 3 reveals peaks of *Alexandrium* abundance and of mollusc toxicity in June, well before the mass mortality event in August. In an advective environment such as the St. Lawrence Estuary, it is likely that this June bloom was dispersed and advected outside of the Estuary well before the August event (as confirmed by our initial modelling work; data not shown here). Accordingly, the mussel toxicity decreased rapidly after the peak in June reaching low values in July. Nevertheless, we can’t exclude that this June event could have contributed, to a certain degree, to seeding of *A*. *tamarense* cells in the St. Lawrence Estuary and to toxicity of marine fauna prior the major bloom and mass mortalities events in August.

Focusing on the August event, our model simulation was initiated with a bloom of 600 km^2^ area in size at Tadoussac on August 4 based on our helicopter survey observations. In our modelling, the bloom at Tadoussac then drifted towards Bic Island on August 5 and 6 (Fig 2A, Site 3), where its position was confirmed by mollusc toxicity (Fig 3). From August 6 to 14, constant winds from the north-east (Fig 1) confined the bloom to the coast. From August 14, winds were calm and the bloom drifted only by tides and freshwater outflow towards the GSL as shown by the arrows on Fig 2. On August 19, strong southerly winds (Fig 1) pushed the bloom offshore and outside of the SLE contributing to its dispersal north-eastward (Fig 2A) before reaching Mont-Louis (Site 5), where *A*. *tamarense* abundances remained weak (Fig 3).

Shellfish rapidly bioaccumulate PST and are often used as sentinel species in toxin monitoring programs. Live blue mussels (*Mytilus edulis*) collected during the event and tested by the AOAC mouse bioassay, revealed extremely high PST (Fig 3) especially near Bic Island (up to 1 ×10^4^ μg STXeq 100 g^−1^ soft tissues, Fig 1D) where the bloom remained for several days (Fig 2A, Site 3). The maximum was 125 times the level considered hazardous for human consumption (80 μg STXeq 100 g^−1^ [26]) and was the highest ever recorded in the SLE since shellfish monitoring began in 1942, attesting to the magnitude and persistence of the toxic *A*. *tamarense* bloom.

During the bloom, carcasses of 10 beluga (*Delphinapterus leucas*) (including 1 on September 10), 7 harbour porpoises (*Phocoena phocoena*) and 85 seals were reported. A dead juvenile fin whale (*Balaenoptera physalus*), a species designated at risk, was also observed drifting on September 17. The number of seal and beluga mortalities was well above the average for the month of August. For example, for the SLE beluga, an endangered population, the 25-year mean number of carcasses for the month of August is 2.6 (S1 Fig). Grey seals (*Halichoerus grypus*) were the most numerous marine mammal found dead, predominantly adult females (20/25 examined), 14 of which were pregnant when examined at necropsy. In addition, 76 reports of mortality events involving hundreds of fish- and mollusc-eating birds belonging to 15 different species, as well as fish and invertebrates, were documented during the month of August (Table 1, Fig 2). Additionally a total of 591 carcasses of birds were observed during a helicopter survey. Most birds (82%) found dead were larids, especially Black-legged Kittiwake (*Rissa tridactyla*, 59%). Other dead birds included Northern Gannet (*Morus bassanus*, 7%), Double-crested Cormorant (*Phalacrocorax auritus*, 4%), alcids (Black Guillemot, *Cepphus grille*, Common Murre, *Uria aalge*, and Razorbill, *Alca torda*, all ≤1.4%), loons (Common Loon, *Gavia immer*, and Red-throated Loon, *Gavia stellata*, each <1%), Common Eider (*Somateria mollissima*,<1%), and Northern Fulmar (*Fulmarus glacialis*,<1%). Sixteen birds were also observed moribund during this survey, 10 of which were larids. Bird mortalities were likely underestimated as numerous carcasses were obscured by algae or debris.

**Table 1:** Concentrations of paralytic shellfish toxins (PST) in tissues of dead specimens collected on beaches or drifting. Diet: major diet. B: birds, F: fish, M: molluscs, Ma: other macro-invertebrates, N: necrophage, Pl: plankton. For more details of specific tissues sampled see Extended Data S1–S3 Tables. % +: Percentage of individuals (pool) which tested positive to STX, nd: not detected, (HPLC): Results from High Performance Liquid Chromatography, COD PST likelihood: number of animals/total examined at necropsy for which cause of death (COD) suspected to be PST based on case definition of laboratory documentation of exposure to the toxin, evidence of acute death (good body condition, food in stomach) and the absence of other significant pathologies.

Species: Group Common name *Latin name* (Diet)	Individuals (pool) tested by ELISA:		Gastro-Intestinal & Contents			Liver/hepatopancreas			Other tissues			COD PST likelihood
			PST : μg STXeq/100g	Samples tested by ELISA :		PST : μg STXeq/100g	Samples tested by ELISA :		PST : μg STXeq/100g	Samples tested by ELISA:		
	N	% +	ELISA (HPLC)	N	% +	ELISA (HPLC)	N	% +	ELISA (HPLC)	N	% +	
**Crustaceans**												
rock crab *Cancer irroratus* (Ma, N)	4	100	6	1	100	37	1	100	nd-13	5	67	
**Molluscs**												
waved whelk *Buccinum undatum* (N)	1	100	83	1	100				9	1	100	
**Fish**												
Atlantic sturgeon *Acipenser oxyrhynchus (*M, Ma)	1	100	33 (24)	1	100	11	1	100				1/1
rainbow smelt *Osmerus mordax* (F, Pl)	6(114)	50	nd-10 (0.2–23)	6	50				nd	6	0	1/1
sand lance *Ammodytes* spp. (Pl)	8(53)	100	93–1010 (1922)	2	100	56-113(435)	2	100	18–1321	5	100	
**Birds**												
Black Guillemot *Cepphus grylle* (F, M, Ma)	6	100	6–70 (14)	6	100	nd-41	6	33				4/4
Black-Legged Kittiwake *Rissa tridactyla* (F, M, Ma)	38	76	nd-134 (80)	19	74	nd-9	20	20	nd-52	17	88	23/32
Bonaparte’s Gull *Larus philadelphia* (F, Ma)	1	0	nd (3)	1	0							1/1
Common Eider *Somateria mollissima* (F, M, Ma)	3	67	6–74	3	67	nd	3	0				1/3
Common Loon *Gavia immer (*B, F, Ma)	2	100	5(2)	1	100	8	2	50				1/1
Common Murre *Uria aalge* (F)	1	0	nd	1	0	nd	1	0				
Double-Crested Cormoran *Phalacrocorax auritus* (F)	13	77	nd-37 (3)	10	70	nd-6	9	22				6/11
Great Black-Backed Gull *Larus marinus* (B, F, Ma)	1	0	nd	1	0	nd	1	0				
Great Blue Heron *Ardea herodias* (F, Ma)	1	0	nd	1	0	nd	1	0				
gull unidentified (F)	4	75	5–74	3	100	34	4	25				1/1
Herring Gull *Larus argentatus* (B, F, M, Ma)	8	88	5–69 (17)	5	100	10	7	14				
Northern Fulmar *Fulmarus glacialis* (F, Ma)	1	0	nd	1	0	nd	1	0				
Northern Gannet *Morus bassanus* (F, Ma)	11	73	11–85 (18)	5	100	85	11	9	nd-9	10	40	4/4
Razorbill *Alca torda* (F)	5	80	nd-71 (96)	4	75	nd-15(6)	4	50				4/5
Red-throated Loon *Gavia stellate* (F, Ma)	1	100	6	1	100	nd	1	0				1/1
Ring-Billed Gull *Larus delawarensis* (F, Ma)	2	100	42	2	100	nd	2	0				2/2
**Mammals**												
beluga *Delphinapterus leucas* (F, Ma)	7	57	nd-63 (0.3–52)	11	36	3 (42–112)	5	20	nd-7 (0.3–16)	7	43	1/2
fin whale *Balaenoptera physalus* (F, Pl)	1	0	nd (0.8–184)	2	0	nd (171)	1	0				1/1
grey seal *Halichoerus grypus* (F, Ma)	24	71	nd-467 (5–16)	17	29	nd-18 (5–9)	20	50	nd-39 (9)	26	35	12/21
grey seal fetus *Halichoerus grypus* (-)	8	50				nd-9	8	50	11	8	12	
harbour porpoise *Phocoena phocoena* (F, Ma)	3	67	nd-7 (3–4)	5	60	nd	3	0				2/3
harbour seal *Phoca vitulina* (F, Ma)	4	75	4–21	4	100	nd-7	4	75	nd-42	4	50	3/4

The sequence of faunal mortalities followed the drift of the bloom along the south coast of the SLE (Fig 2). Three days after the initiation of the bloom, about 100 dead or moribund birds (8 species) were first observed near Tadoussac (Fig 2A, Site 1) by Parks Canada staff. Over the subsequent 20 day period, the area with reported mortalities progressively expanded eastward along the Gaspé Peninsula following the drift of the bloom. Following dispersal of the bloom around August 21 near Sainte-Anne-des-Monts, mammal carcasses continued to be reported for a few more days but most were decomposed. Few carcasses were found outside the zone affected by the *A*. *tamarense* bloom. For example, some were reported near Baie-Comeau (Fig 2A, Site 9) coinciding with a localized small increase in *A*. *tamarense* abundance and shellfish toxicity (Fig 3). Five dead Northern Gannets were also found in the GSL from August 9 to 15. Adult gannets on GSL breeding colonies are feeding their young chicks in August and adults often forage for fish in the SLE to feed themselves and their chicks [27].

**Fig 4:**
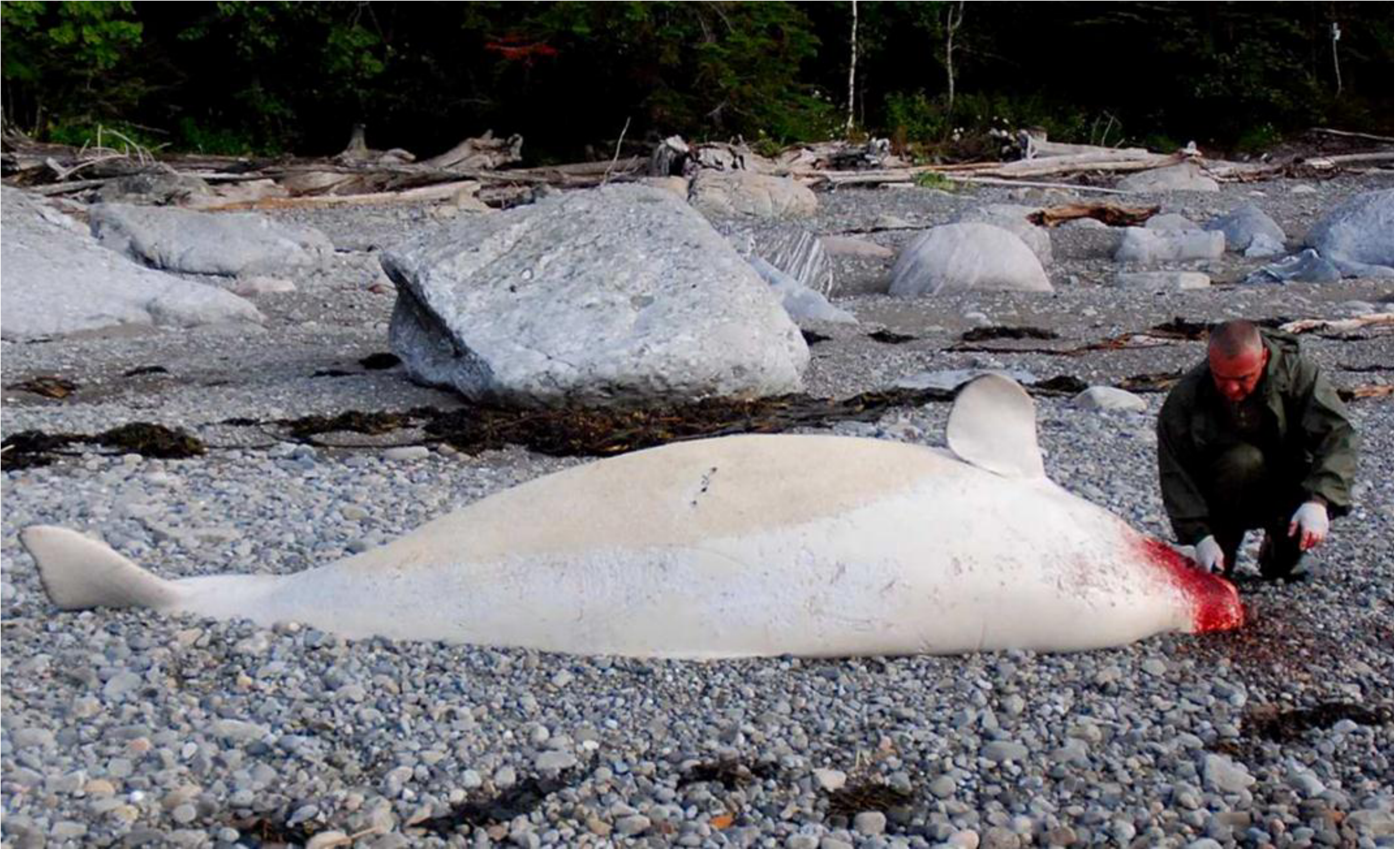
Observed gross lesions consistent with PST. Adult, 22 year old, female beluga found dead in the SLE during the *A*. *tamarense* bloom. Note blood on the skin of the head. Positive for PST in stomach (63.2 μg 100 g^-1^) and kidney (7.3 μg 100 g^-1^) by ELISA and HPLC. Liver tested positive for PST by HPLC.

Pathological analyses were performed on a total of 74 birds of 13 species, 10 fish of 2 species, 21 grey seals, 4 harbour seals, 3 harbour porpoises, 2 beluga and 1 fin whale. Carcass preservation was evaluated as good (fresh/edible) in 32% of cases, fair (decomposed, but organs basically intact): 37%, and poor (advanced decomposition): 31%. Decomposition may have influenced detection of toxins as the tissues became friable and liquefied, exposing toxins to intense enzymatic and microbial degradation (see below). Most birds (67%) and almost all marine mammals (96%) examined at necropsy were in good nutritional condition and many with food in stomachs. Pathological analyses did not identify a cause of death in 85% of cases (i.e., gross lesions could not be attributed to an etiological agent such as a pathogen, traumatic injury or other specific disease process) (Table 1). Gross lesions included wet, heavy and congested lungs likely due to respiratory paralysis consistent with PST [1]. Congestion of the tracheal and oral mucosa was also observed. Some of the intoxicated grey seals and one beluga had blood-stained fur or skin on the head, possibly caused by irritation and motor incoordination due to PST [1] (Fig 4). One of the intoxicated beluga had superficial parallel cutaneous lacerations associated with haemorrhage in subcutaneous tissues on its flank highly suggestive of boat propeller trauma (S2 Fig). Animals paralysed due to PST may be more vulnerable to vessel collisions.

Various tissues from carcasses were tested for PST by Enzyme-Linked Immunosorbent Assay (ELISA), with selected samples analysed by LC-MS and pcox-FLD (Table 1, see also S1–S3 Tables). Analyses revealed levels of PST above detection limits (i.e., >2.2 μg 100 g^-1^) in the liver and/or gastrointestinal tract (GIT) contents (Table 1) as well as other tissues (S1–S3 Tables) in more than half of the 321 carcasses tested. An unidentifiable fish from the stomach of a Razorbill, which tested positive for saxitoxin in the liver (11 μg 100 g^-1^) and GIT tissues (64.1 to 71.2 μg 100 g^-1^), also tested positive for saxitoxin (60 μg 100 g^-1^), providing strong evidence for the trophic transfer of PST leading to mortality. Of 8 grey seal fetuses examined, 4 tested positive for PST. Liver and brain tissues of 6 of the corresponding 8 pregnant females tested positive for PST indicating transplacental transfer.

Limited diet data from marine birds and mammals in the SLE indicate that harbour seals, porpoises, razorbills, kittiwakes, gannets, and cormorants feed commonly on coastal planktivorous fish such as sand lance (*Ammodyte*s sp.) and capelin (*Mallotus villosus*) [27–31]. Cod (*Gadus morhua*) and herring (*Clupea harengus*) are important prey for grey seals in August [32]. Beluga are generalists, feeding on sand lance, redfish (*Sebastes* spp.), capelin, herring and some benthic invertebrates [28]. Some rock crabs (*Cancer irroratus*) found dead are necrophagic. The presence of PST in live planktivorous and higher trophic level fish as well as in benthic invertebrates and zooplankton samples collected during the bloom (S4 and S5 Tables) provides direct evidence for the trophic transfer of PST leading to mortality. Higher mortalities among female vs. male grey seals, beluga and porpoises may indicate differences in prey preferences or foraging behaviour, seasonal geographic segregation of the sexes [33, 34] or a differential response to biotoxins due to differences in body mass or physiology [35].

**Fig 5:**
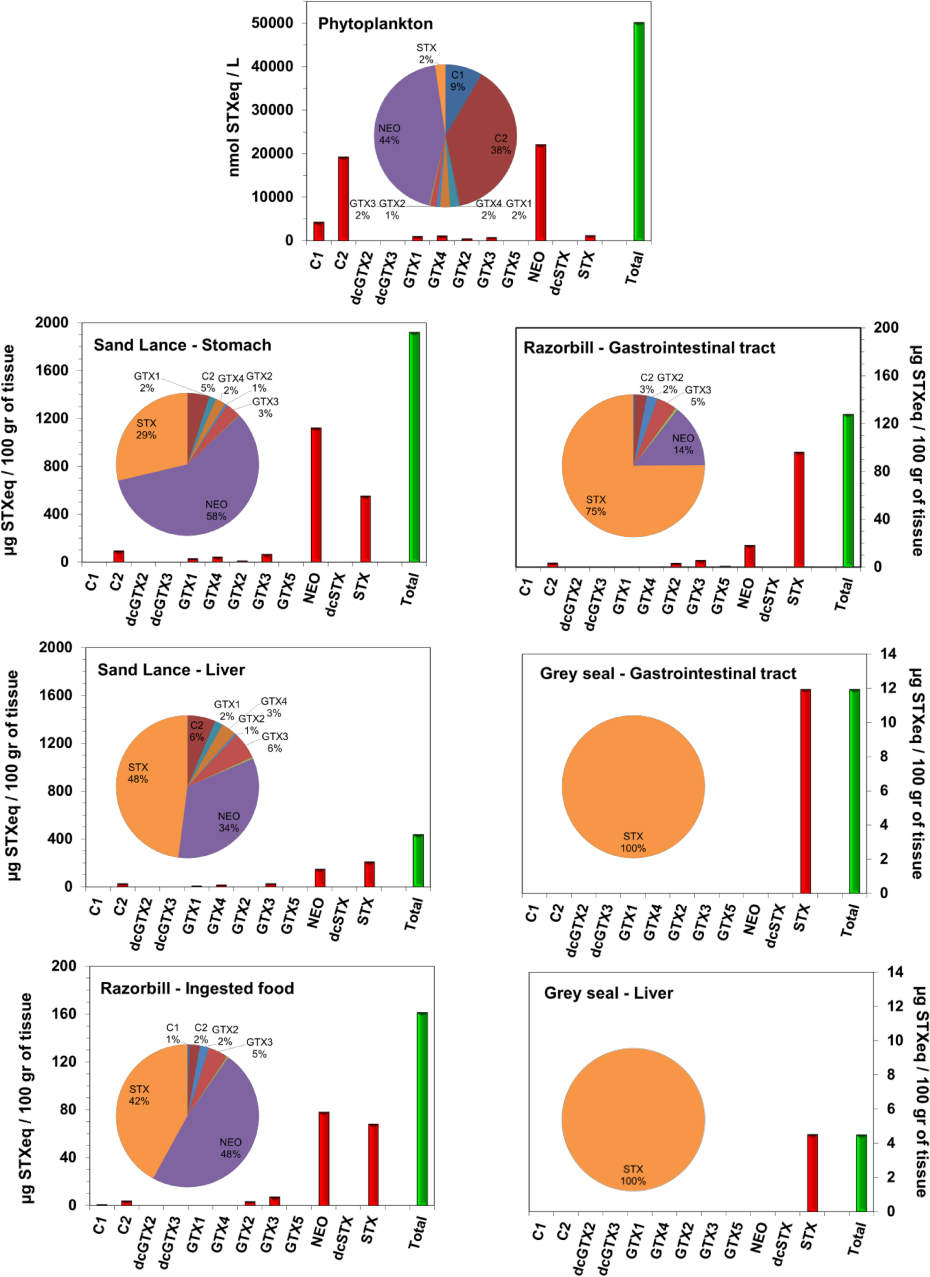
Modifications of toxin profile through food web. Typical toxin profile (in STX equivalents and in % of the total toxicity) determined by LC-MS and LC-pcox-FLD of phytoplankton samples and in the liver and/or stomach, gastrointestinal tract/contents from selected fish, bird and mammal carcasses.

PST exists as a suite of over 21 related molecular forms that vary in toxicity, with saxitoxin (STX) being the parent form and one of the most toxic [1, 11]. The precise mixture (profile) of toxins varies depending on the strain of *Alexandrium* spp. as well as on metabolic transformation by the consumer from lower to higher trophic levels and its state of degradation [36–38]. Toxin profiles derived from HPLC are shown in phytoplankton, sand lance (stomach and liver), Razorbill (stomach contents and GIT) and grey seal (GIT and liver), all collected during the bloom (Fig 5). The phytoplankton contained principally neosaxitoxin (NEO), N-sulfogonyautoxin-2 and -3 (C1 and C2), with smaller amounts of gonyautoxin-1 to -4 (GTX1-4) and STX. In sand lance, STX became relatively more abundant. In Razorbill the profile was characterized by NEO and STX with other toxins declining in importance. The profile in the unidentifiable fish from the stomach of the Razorbill resembled that of sand lance, while STX dominated in the Razorbill’s GIT. In grey seal, the profile was composed entirely of STX. Some studies have reported similar shifts in profile after consumption of algae by shellfish, finfish and higher animals [36–38]. Transformation of C and GTX analogs to NEO and STX, as well as NEO to STX, have been reported and can be the result of chemical, bacterial and enzymatic actions [39–42]. There have been no studies, to our knowledge, of metabolism in mammalian species but it is reasonable to assume that transformations can continue as the toxins go up through higher trophic levels in the food web.

Additional evidence of exposure to PST comes from reports of apparently neurologically impaired fauna exhibiting unusual behaviour. These include paralysed and uncoordinated sand lance, observed by a DFO SCUBA diver at 2.4 m depth as the bloom drifted past Sainte-Flavie (Fig 2A, Site 4) on August 15; gulls, cormorants and eiders, unaware of their surroundings, unable to enter the water, to raise their head above the water, or to flee a helicopter hovering low above the beach; freshly wounded, sick or orphaned marine mammals, some exhibiting erratic behaviour, including 2 beluga, 1 minke whale (*Balaenoptera acutorostrata*), 1 grey seal and 2 unidentified seals.

Although it is difficult to demonstrate cause of death due to PST, the following evidence supports the conclusion of a multispecies mortality event due to a toxic *A*. *tamarense* bloom: a) PST detected in plankton samples; b) elevated PST levels in molluscs; c) the spatio-temporal occurrence of mortalities following the drift of the bloom, with d) no other cause of death identified pathologically and with PST detected in tissues from these animals; and e) signs of neurological dysfunction and acute death (good body condition, food in stomach) consistent with PST intoxication. Until now, reports of mass mortalities as a result of PST-producing algal blooms have often been anecdotal and many of these remain unpublished. The event reported here is the first well-documented case of mass mortality of multiple species of marine fauna resulting from an *Alexandrium* bloom. Such mortalities are expected to increase in the future as the frequency, intensity and geographic extent of toxic algal blooms are apparently increasing world-wide to climate change, coastal eutrophication and other environmental perturbations [43, 44].

## Materials and methods

### Ethics statement

Field permit: Phytoplankton data come from the long-term monitoring program of the Department of Fisheries and Oceans Canada (DFO). Live invertebrates and fish specimens were collected by DFO staff or with permission of this department. No permit is required in Canada to collect marine fauna carcasses on beaches or drifting, nor for necropsy of carcasses.

Animal research: Marine birds and mammals, including species designated at risk were examined at necropsy only after their natural death.

Fig 4: The participant on this figure has given written informed consent (as outlined in PLOS consent form) to publish these case details.

### Toxic algae count and identification

As part of the DFO toxic algae monitoring program, phytoplankton samples were routinely collected at 11 coastal sites on weekly basis from May to October 2008. Sea surface (<1 m) phytoplankton samples were collected with a Niskin bottle or a bucket and preserved with Lugol’s iodine solution (1% final concentration). Subsamples (100 ml) were settled (Utermöhl technique) and toxic algae counted with an inverted microscope [45] by experienced taxonomists using [46] as the taxonomy guide. *A*. *tamarense* cell counts included in the present study are from samples (n = 137) collected between 23 May and 23 September 2008 at 6 selected monitoring sites (Tadoussac, Sainte-Flavie, Mont-Louis, Port-Daniel/Gascons, Baie-Comeau, Sept-Îles) (Fig 3). *A*. *tamarense* was the only toxic species present in quantity to explain this mass mortality.

### Model simulation, atmospheric and meteorological data

The trajectory of the dinoflagellate bloom was calculated as follows. Daily forecasts of surface currents in the SLE are calculated daily and posted at http://slgo.ca/ocean/index.jsp?lg=en. These forecasts are calculated using the application of Saucier et al. [47–49], a 3-D circulation model on a 5 km grid resolution. The model is driven by freshwater run off, monthly mean value at Quebec City and from coastal rivers, tides at the straits of Belle-Isle and Cabot, and by atmospheric forcing: air temperature, wind intensity, dew point, cloud cover, precipitation and evaporation, provided by Environment Canada (http://www.weatheroffice.gc.ca/canada_e.html). Since the top layer in the model is 5 m thick, wind induced surface currents are underestimated in the model. To reproduce surface trajectories of either an oil spill or a phytoplankton bloom, a surface velocity is added to the forecasted currents using 3% of wind intensity in the direction of the wind. The trajectory of the dinoflagellate bloom was calculated in the field of surface currents using a 4^th^ order Runge-Kutta interpolator. This trajectory was updated daily using either the end point of the previous simulation or from direct observation confirming the location of the bloom. Simulations were initiated at site 1 with a bloom size of 600 km^2^ which was estimated from a helicopter survey (see below).

### AOAC mouse bioassay

Data for saxitoxins in commercial shellfish are collected as part of ongoing monitoring efforts performed routinely by the CFIA. Over 100 sampling sites distributed along the coastline of the Estuary and Gulf of St. Lawrence are used to collect various species of bivalve shellfish. In 2008, shellfish samples were analyzed for PSP toxins using the standard mouse bioassay, and collection and toxin analytical methods were performed according to the AOAC protocol [50]. The results of mouse bioassays were converted to toxicity units: μg saxitoxin equivalents (STXeq) kg^−1^ wet weight of edible mollusc tissue [50]. Toxin data included in the present study are from samples (n = 718) collected between 23 May and 23 September 2008 at 8 selected monitoring sites (Fig 3), which were sampled on weekly basis prior, during, and following the red tide. Only the data coming from blue mussels *Mytilus edulis* and softshell clam *(Mya arenaria)* were considered in the present study although some other additional marine invertebrate species were also analysed during the red tide event by the AOAC mouse bioassay.

### Helicopter surveys

Between August 13 and 16, 2008, a survey was conducted by helicopter equipped with bubble windows in order to quantify, locate and identify dead or moribund birds. Two experienced observers from CWS noted all sightings using PC-Mapper geo-referenced voice recording software (Corvallis Microtechnology, Corvallis, Oregon, USA). The actual survey time totalled 13.5 h and covered 1193 km around islands and coastal areas of the SLE, between Kamouraska and Sainte-Anne-des-Monts (south shore) and between Baie-Trinité and Baie-Saint-Paul (north shore). A second helicopter survey was also performed between August 14 and 15, 2008, in the same region in order to locate the bloom. This survey allows us to estimate visually the size of the bloom from discoloration of waters.

### Necropsies and pathological analyses

Necropsies and pathological analyses were performed by or under the supervision of veterinary pathologists. Carcasses were examined either fresh or after one cycle of freezing and thawing. For marine mammals, bird and fish, the stage of decomposition of specimens was quantified by the code system established by Geraci and Lounsbury [51]. Thus, each carcass examined was assigned to one to five preservation categories determined by several characteristics such as described in [51]: CODE 1: Live Animals; CODE 2: Carcass in Good Condition (Fresh/Edible); CODE 3: Fair (Decomposed, but organs basically intact); CODE 4: Poor (Advanced decomposition); and CODE 5: Mummified or Skeletal Remains. Age of marine mammals was also determined using sectioned teeth [52]. At necropsy, nutritional states of specimens were visually evaluated [52]. Essentially, animals in good flesh with no evidence of muscular or fat depletion due to mobilization of protein or fat reserves (i.e., animals are not emaciated suggestive of starvation) were considered as in good nutritional condition. Gross lesions of dead organisms were also noted and multiple samples, including stomach contents when present, were collected. Tissues of major organs (lung, kidney, gonads, mammary gland, uterus) or suspected were processed for histopathological evaluation by light microscopy using standard laboratory procedures to detect any lesions or abnormal tissue [52]. Ancillary tests, including aerobic microbiological culture were conducted as needed based on histopathological findings in order to identify any pathogens (bacteria, virus, fungus, and parasite) present in tissues [52].

### Collection of live invertebrates and fish

Specimens were obtained from the DFO research trawler Teleost and commercial fishing boats between August 8 and 27, 2008. Collections were made at depths ranging from 65 to 315 m between 49° 04.4' N, 67° 11.9' W and 48° 34.9' N, 68° 35.9' W. Zooplankton were collected using a 0.75 m ring net (202 μm mesh) towed vertically from bottom to surface at 1 m s^-1^. Specimens were immediately frozen for later quantification of PST (see below).

### PST assays

Tissues were assayed using ELISA for saxitoxin (Abraxis LLC, Warminster, PA, USA). Samples, standards and controls were processed according to the ELISA kit instructions. However, the extraction protocol was modified to facilitate further testing of selected samples via HPLC, and to prevent the hydrolytic interconversion of toxin congeners, by performing all extractions in 0.1 M acetic acid [53]. Concentrations were calculated against the standard curve response such as described in the ELISA kit instructions.

### Instrumental analysis of PST composition and quantification

Quantitative measurements of toxin composition were performed by liquid chromatography with post-column oxidation and fluorescence detection (HPLC-pcox-FLD) [53], with additional confirmation by HPLC–MS/MS on an API 4000 Q-trap LC-MS (applied Biosystems) with Agilent 1200 HPLC using a triple quadrupole detector and ion-spray [54]. Toxin concentrations were converted [24] to toxicity units using specific toxicity conversion factors provided in Oshima [55].

## Supporting information

S1 Fig Historical beluga mortalities. Mean number of SLE beluga carcasses by month for the period 1983 to 2007 and total each month in 2008.
(TIFF)

S2 Fig Intoxication and vulnerability to vessel collisions. Intoxicated beluga with superficial cutaneous lacerations likely caused by a boat propeller. Positive for PST in GI (8.2 μg 100 g^-1^) by HPLC.
(TIFF)

S1 Table Concentrations of paralytic shellfish toxins (PST) in tissues of dead invertebrates and fish collected on beaches or drifting. Diet key Ag: macroalgae, B: birds, F: fish, M: molluscs, Ma: other macro-invertebrates, N: necrophage, Pl: plankton. Viscera comprise all soft internal organs sampled together. % +: Percentage of individuals (pool) which tested positive to STX, nd: not detected, (HPLC): Results from High Performance Liquid Chromatography, COD PST likelihood: number of animals/total examined at necropsy for which cause of death (COD) suspected to be PST based on case definition of laboratory documentation of exposure to the toxin, evidence of acute death (good body condition, food in stomach) and the absence of other significant pathologies.
(PDF)

S2 Table Concentrations of paralytic shellfish toxins (PST) in tissues of dead birds collected on beaches or drifting. Abbreviation definitions are given in S1 Table.
(PDF)

S3 Table Concentrations of paralytic shellfish toxins (PST) in tissues of dead mammals collected on beaches or drifting. Abbreviation definitions are given in S1 Table.
(PDF)

S4 Table Concentrations of paralytic shellfish toxins (PST) in tissues of live invertebrates. Abbreviation definitions are given in S1 Table.
(PDF)

S5 Table Concentrations of paralytic shellfish toxins (PST) in tissues of live fish. Abbreviation definitions are given in S1 Table.
(PDF)

S1 File Data (including metadata) collected in this study. (7Z)
