# Supplementary figures and images for: Multispecies mass mortality of marine fauna linked to a toxic dinoflagellate bloom
Source: PLoS One. 2017 May 4;12(5):e0176299. doi: 10.1371/journal.pone.0176299 (PMC5417436; doi:10.1371/journal.pone.0176299)

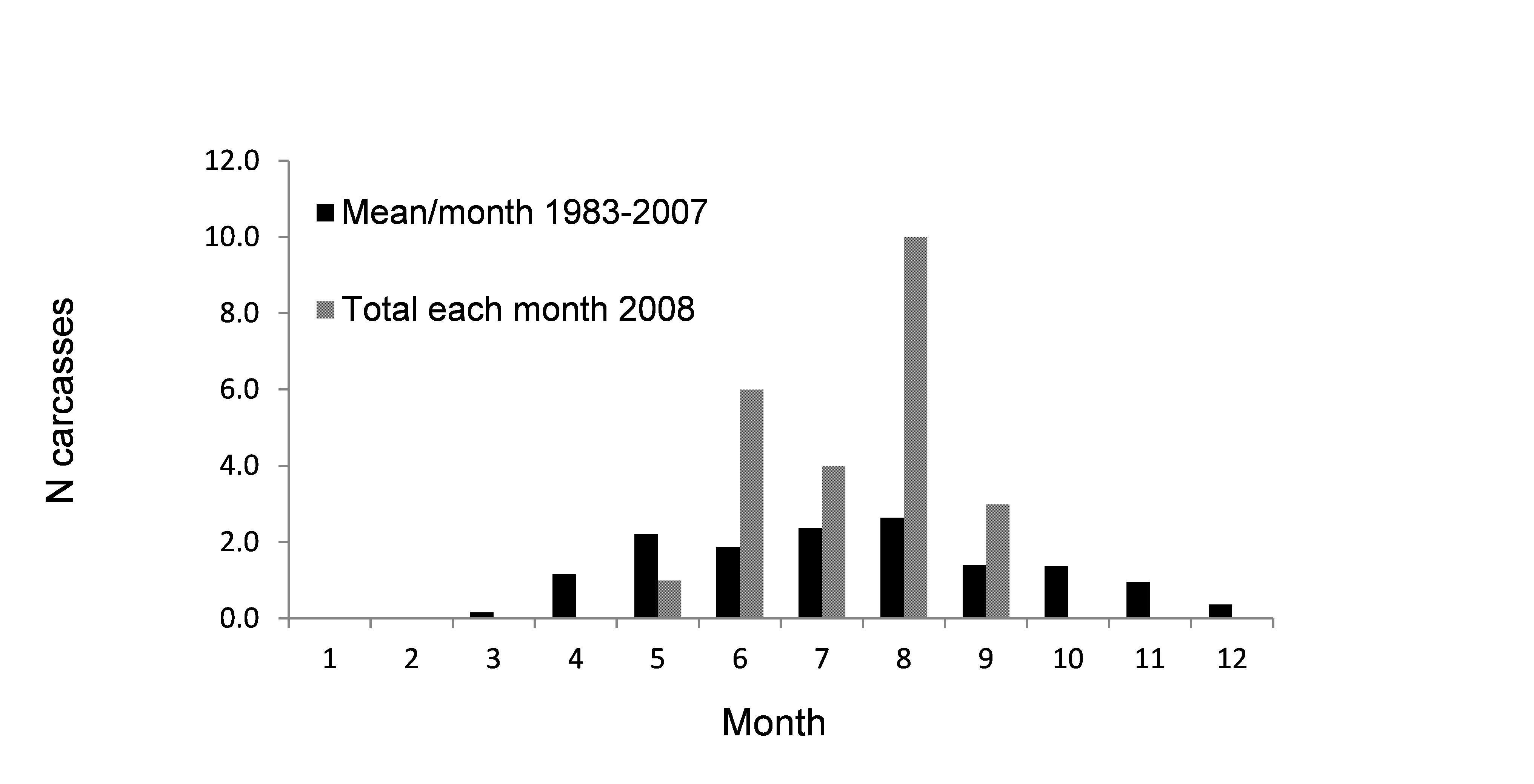

Supplement: S1 Fig — Mean number of SLE beluga carcasses by month for the period 1983 to 2007 and total each month in 2008. (TIFF) [file pone.0176299.s001.tiff]

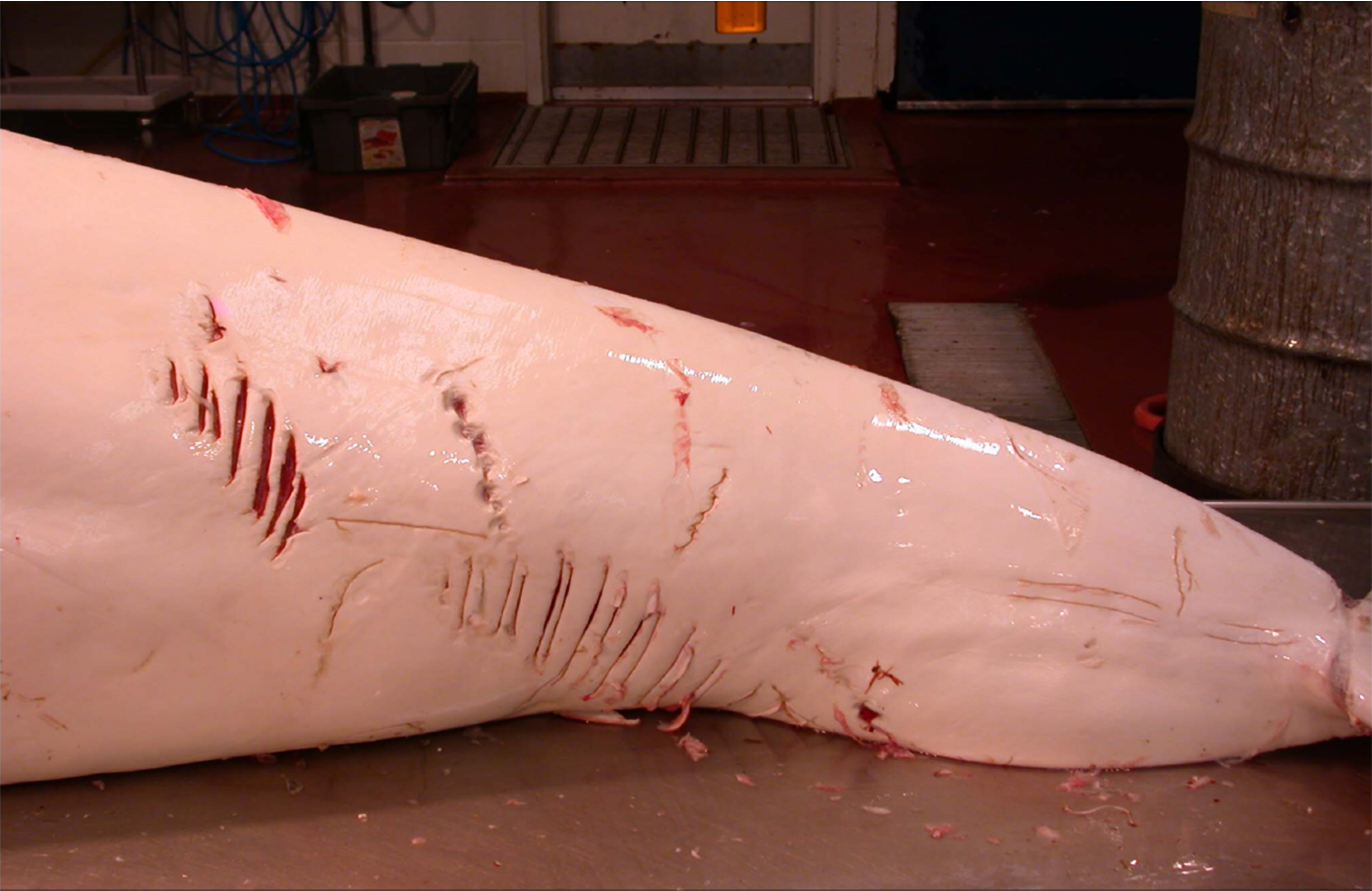

Supplement: S2 Fig — Intoxicated beluga with superficial cutaneous lacerations likely caused by a boat propeller. Positive for PST in GI (8.2 μg 100 g-1) by HPLC. (TIFF) [file pone.0176299.s002.tiff]
