# Supplementary material for: Multispecies mass mortality of marine fauna linked to a toxic dinoflagellate bloom
Source: PLoS One. 2017 May 4;12(5):e0176299. doi: 10.1371/journal.pone.0176299 (PMC5417436; doi:10.1371/journal.pone.0176299)
Supplement: S1 Table — Diet key Ag: macroalgae, B: birds, F: fish, M: molluscs, Ma: other macro-invertebrates, N: necrophage, Pl: plankton. Viscera comprise all soft internal organs sampled together. % +: Percentage of individuals (pool) which tested positive to STX, nd: not detected, (HPLC): Results from High Performance Liquid Chromatography, COD PST likelihood: number of animals/total examined at necropsy for which cause of death (COD) suspected to be PST based on case definition of laboratory documentation of exposure to the toxin, evidence of acute death (good body condition, food in stomach) and the absence of other significant pathologies. (PDF) [file pone.0176299.s003.pdf]

S1 Table. Concentrations of paralytic shellfish toxins (PST) in tissues of dead invertebrates and fish collected on beaches or drifting. Diet key Ag: macroalgae, B: birds, F: fish, M: molluscs, Ma: other macro-invertebrates, N: necrophage, Pl: plankton. Viscera comprise all soft internal organs sampled together. % +: Percentage of individuals (pool) which tested positive to STX, n.d.: not detected, (HPLC): Results from High Performance Liquid Chromatography, COD PST likelihood: number of animals/total examined at necropsy for which cause of death (COD) suspected to be PST based on case definition of laboratory documentation of exposure to the toxin, evidence of acute death (good body condition, food in stomach) and the absence of other significant pathologies.

**Species common name *(Latin name)***	**Major Diet**	**Samples tested by ELISA**		**Corresponding tissues**		**PST concentration (µg/100g)**		**COD PST likelihood**
		**N (indiv.)**	**% +**	**Tissue (n)**	**% +**	**ELISA**	**HPLC**	
**Crustaceans**								
rock crab	Ma, N	4	100	Flesh (2)	50	5.8		
(Cancer irroratus)				Stomach contents (1)	100	5.7		
				Gill (1)	0	n.d.		
				Gonad (1)	100	13.3		
				Hepatopancreas (1)	100	37		
				Viscera (1)	100	9.2		
**Molluscs**								
waved whelk	N	1	100	Flesh (1)	100	8.6		
(Buccinum undatum)				Viscera (1)	100	83		
**Fish**								
sand lance	Pl	8 (53)	100	Gonad (1)	100	270		
(Ammodytes spp.)				Head (3)	100	18-84		
				Viscera (1)	100	1321		
				Stomach contents (1)	100	93	1922	
				Liver (2)	100	56-113	435	
				Digestive tract (1)	100	1010		
rainbow smelt	Pl, F	6 (114)	50	Head (6)	0	n.d.		1/1
(Osmerus mordax)				Viscera (6)	50	n.d.-10	0.2-23	
Atlantic sturgeon	Ma, M	1	100	Stomach contents (1)	100	33	24	1/1
(Acipenser oxyrhynchus)				Liver (1)	100	11		
