# Supplementary material for: Multispecies mass mortality of marine fauna linked to a toxic dinoflagellate bloom
Source: PLoS One. 2017 May 4;12(5):e0176299. doi: 10.1371/journal.pone.0176299 (PMC5417436; doi:10.1371/journal.pone.0176299)
Supplement: S2 Table — Abbreviation definitions are given in S1 Table. (PDF) [file pone.0176299.s004.pdf]

S2 Table. Concentrations of paralytic shellfish toxins (PST) in tissues of dead birds collected on beaches or drifting. Abbreviation definitions are given in S1 Table.

Species common name *(Latin name)*	Major Diet	Samples tested by ELISA		Corresponding tissues		PST concentration (µg/100g)		COD PST likelihood
		N (indiv.)	% +	Tissue (n)	% +	ELISA	HPLC	
**Birds**								
Double-crested Cormorant	F	13	77	Liver (9)	22	n.d-5.8		6/11
(Phalacrocorax auritus)				Digestive tract (10)	70	n.d-37	2.7	
Common Eider	M, Ma, F	3	67	Liver (3)	0	n.d.		1/3
(Somateria mollissima)				Digestive tract (3)	67	n.d-74		
Northern Gannet	F, Ma	11	73	Liver (11)	9	85		4/4
(Morus bassanus)				Digestive tract (5)	100	11-85	18	
				Kidney (5)	40	n.d-6.3		
				Muscle (5)	40	n.d-8.7		
Northern Fulmar	Ma, F	1	0	Liver (1)	0	n.d.		
(Fulmarus glacialis)				Digestive tract (1)	0	n.d.		
gull	F	4	75	Liver (4)	25	33.7		1/1
(not identified)				Digestive tract (3)	100	5.4-74		
Ring-billed Gull	Ma, F	2	100	Liver (2)	0	n.d.		2/2
(Larus delawarensis)				Digestive tract (2)	100	42		
Herring Gull	F, Ma, M, B	8	88	Liver (7)	14	10		
(Larus argentatus)				Digestive tract (5)	100	4.7-69	17	
Great Black-backed Gull	F, Ma, B	1	0	Liver (1)	0	n.d.		
(Larus marinus)				Digestive tract (1)	0	n.d.		
Great Blue Heron	F, Ma	1	0	Liver (1)	0	n.d.		
(Ardea herodias)				Digestive tract (1)	0	n.d.		
Black Guillemot	Ma, F, M	6	100	Liver (6)	33	n.d.-41		4/4
(Cepphus grylle)				Digestive tract (6)	100	6.4-70	14	
Common Murre	F	1	0	Liver (1)	0	n.d.		
(Uria aalge)				Digestive tract (1)	0	n.d.		
Black-legged Kittiwake	Ma, M, F	38	76	Liver (20)	20	n.d-8.8		23/32
(Rissa tridactyla)				Digestive tract (19)	74	n.d.-134	80	
				Digestive tract & liver (17)	88	n.d.-52		
Bonaparte's Gull	Ma, F	1	0	Digestive tract (1)	0	n.d.	3.1	1/1
(Larus philadelphia)								
Razorbill	F	5	80	Liver (4)	50	n.d.-15	5.8	4/5
(Alca torda)				Digestive tract (4)	75	n.d.-71	96	
Red-throated Loon	Ma, F	1	100	Liver (1)	0	n.d.		1/1
(Gavia stellate)				Digestive tract (1)	100	6.1		
Common Loon	Ma, F, B	2	100	Liver (2)	50	7.7		1/1
(Gavia immer)				Digestive tract (1)	100	4.5	1.9	
