# Supplementary material for: Multispecies mass mortality of marine fauna linked to a toxic dinoflagellate bloom
Source: PLoS One. 2017 May 4;12(5):e0176299. doi: 10.1371/journal.pone.0176299 (PMC5417436; doi:10.1371/journal.pone.0176299)
Supplement: S3 Table — Abbreviation definitions are given in S1 Table. (PDF) [file pone.0176299.s005.pdf]

S3 Table. Concentrations of paralytic shellfish toxins (PST) in tissues of dead mammals collected on beaches or drifting. Abbreviation definitions are given in S1 Table.

Species common name *(Latin name)*	Major Diet	Samples tested by ELISA		Corresponding tissues		PST concentration (µg/100g)		COD PST likelihood
		N (indiv.)	% +	Tissue (n)	% +	ELISA	HPLC	
**Mammals**								
beluga	F, Ma	7	57	Stomach contents (4)	50	n.d.-63	0.3-52	1/2
(Delphinapterus leucas)				Liver (5)	20	2.7	42-112	
				Kidney (6)	50	n.d.-7.3	0.3-16	
				Blood (1)	0	n.d.	4.3	
				Feces (2)	50	n.d.-57	16-50	
				Intestine (3)	0	n.d.	8.2	
				Intestinal contents (2)	50	3.5		
harbour porpoise	F, Ma	3	67	Liver (3)	0	n.d.		2/3
(Phocoena phocoena)				Stomach contents (3)	67	n.d.-6.5	3.4	
				Intestine (2)	50	6.1	4.4	
fin whale	Pl, F	1	0	Liver (1)	0	n.d.	171	1/1
(Balaenoptera physalus)				Stomach contents (1)	0	n.d.	184	
				Intestine (1)	0	n.d.	0.8	
harbour seal	F, Ma	4	75	Liver (4)	75	n.d.-7		3/4
(Phoca vitulina)				Stomach contents (3)	100	4-11		
				Kidney (1)	100	8.8		
				Blood (1)	0	n.d.		
				Lung (1)	0	n.d.		
				Bladder (1)	100	42		
				Feces (1)	100	21		
grey seal	F, Ma	24	71	Liver (20)	50	n.d.-18	4.5-9.4	12/21
(Halichoerus grypus)				Stomach contents (15)	20	n.d.-467	4.9-16	
				Kidney (7)	29	n.d.-9.2		
				Blood (8)	0	n.d.	8.8	
				Lung (7)	43	n.d.-4.5		
				Feces (2)	100	27-35		
				Bladder (4)	100	17-39		
grey seal	-	8	50	Liver of fetus (8)	50	n.d.-8.9		
(Halichoerus grypus)				Blood of fetus (8)	12	11		
