# Supplementary material for: Multispecies mass mortality of marine fauna linked to a toxic dinoflagellate bloom
Source: PLoS One. 2017 May 4;12(5):e0176299. doi: 10.1371/journal.pone.0176299 (PMC5417436; doi:10.1371/journal.pone.0176299)
Supplement: S4 Table — Abbreviation definitions are given in S1 Table. (PDF) [file pone.0176299.s006.pdf]

S4 Table. Concentrations of paralytic shellfish toxins (PST) in tissues of live invertebrates.

Abbreviation definitions are given in S1 Table.

**Species common name** *(Latin name)*	**Major Diet**	**Samples tested by ELISA**		**Corresponding tissues**		**PST concentration (µg/100g)**	
		**N (indiv.)**	**% +**	**Tissue (n)**	**% +**	**ELISA**	**HPLC**
**Zooplankton**	Pl	19	26	Whole (many)		9.6-76	6-71
**Crustaceans**							
copepod	Pl	1 (20)	0	Whole (1)	0	n.d.	
(Calanus finmarchicus)							
northern shrimp	Pl		67	Flesh and intestine (8)	25	n.d.-5.2	
(Pandalus borealis)				Flesh only (3)	0	n.d.	
				Gonad and viscera (7)	86	n.d.-8.1	
				Head (6)	50	n.d.-6.8	
				Whole (1)	100	56	
**Echinoderms**							
deep-sea heart urchin	N	3 (24)	0	Various tissues (3)	0	n.d.	
(Brisaster fragilis)							
green urchin	Ag	7 (52)	14	Body cavity fluid (7)	14	4.1	
(Strongylocentrotus droebachiensis)							
**Molluscs**							
waved whelk	N	15 (41)	87	Flesh (8)	25	n.d.-4.8	
(Buccinum undatum)				Liver (4)	100	43-900	
				Viscera (10)	100	4.5-74	
Nuttall's cockle	Pl	3 (5)	100	Flesh (1)	0	n.d.	
(Clinocardium nuttallii)				Viscera (1)	100	8.1	
				Whole (2)	100	5.0-5.6	
colus whelk	N	1	100	Flesh (1)	0	n.d.	
(*Colus* sp.)				Viscera (1)	100	8.8	
broad yoldia	Pl	2 (3)	100	Whole (2)	100	71-116	
(Megayoldia thraciaeformis)							
mussel	Pl	2	100	Whole (2)	100	10-43	
(*Mytilus* sp.)							
rejected neptune	N	2	100	Viscera (1)	100	19	
(Neptunea despecta)				Whole (1)	100	> 144	
