# Supplementary material for: Multispecies mass mortality of marine fauna linked to a toxic dinoflagellate bloom
Source: PLoS One. 2017 May 4;12(5):e0176299. doi: 10.1371/journal.pone.0176299 (PMC5417436; doi:10.1371/journal.pone.0176299)
Supplement: S5 Table — Abbreviation definitions are given in S1 Table. (PDF) [file pone.0176299.s007.pdf]

S5 Table. Concentrations of paralytic shellfish toxins (PST) in tissues of live fish. Abbreviation definitions are given in S1 Table.

Species common name *(Latin name)*	Major Diet	Samples tested by ELISA		Corresponding tissues		PST concentration (µg/100g)	
		N (indiv.)	% +	Tissue (n)	% +	ELISA	HPLC
**Fish**							
hookear sculpin	Ma, M	1	0	Various tissues (1)	0	n.d.	
(Artediellus sp.)							
capelin	Pl	6 (83)	100	Flesh (4)	25	4	
(Mallotus villosus)				Heart liver & kidney (4)	25	4.1	
				Digestive tract & gonad (6)	100	4.6-12	
fourline snakeblenny	Ma	1 (3)	100	Flesh (1)	0	n.d.	
(Eumesogrammus praecisus)				Viscera (1)	0	n.d.	
				Head (1)	100	6.5	
Atlantic herring	Pl	3 (5)	33	Flesh (3)	0	n.d.	
(Clupea harengus)				Gonads (3)	0	n.d.	
				Stomach & intestine (3)	33	n.d.-20	
rock gunnel	M, Ma	1	0	Various tissues (1)	0	n.d.	
(Pholis gunnellus)							
sand lance	Pl	2	50	Whole (1)	0	n.d.	
(Ammodytes spp.)				Flesh (1)	100	4.8	
				Head and viscera (1)	100	10	
sculpin		2	0	Various tissues (2)	0	n.d.	
(not identified)							
rainbow smelt	Pl, F	6 (many)	17	Whole (1)	100	5.1	
(Osmerus mordax)				Flesh (3)	0	n.d.	
				Head (2)	0	n.d.	
				Viscera (5)	0	n.d.	
Atlantic mackerel	Pl, F	2	100	Flesh (2)	0	n.d.	
(Scomber scombrus)				Liver (2)	100	109-1634	
				Gonad (2)	100	58-70	
				Digestive tract (2)	100	27-55	
Greenland halibut	F, Ma	5	0	Various tissues (5)	0	n.d.	
(Reinhardtius hippoglossoides)							
American plaice	Ma, M, F	7 (17)	0	Various tissues (7)	0	n.d.	
(Hippoglossoides platessoides)							
fourbeard rockling	Ma	4 (15)	25	Flesh (4)	25	4.2	
(Enchelyopus cimbrius)							
